# No germline mutations in *CDKN2A* (*p16*) in patients with squamous cell cancer of the head and neck and second primary tumours

**DOI:** 10.1054/bjoc.2001.2068

**Published:** 2001-09-01

**Authors:** S Jefferies, S M Edwards, R A Hamoudi, R A'Hern, W Foulkes, D Goldgar, M. P. T. Collaborators, R Eeles

**Affiliations:** Cancer Genetics, Institute of Cancer Research, 15 Cotswold Rd, Sutton, Surrey; The Royal Marsden Hospital Trust, Downs Rd, Sutton, Surrey, SM2 5PT; McGill University, Montreal, Canada; IARC, Rickmansworth Road, Lyon, France; Guys and St Thomas' and Kings Hospital Trusts; Mount Vernon Hospital Trust, Northwood, Middlesex, HA6 2RN; Mid-Kent Oncology Centre, Hermitage Lane, Maidstone, Kent, ME16 9QQ; Queen Victoria Hospital, East Grinstead; Royal Surrey County Hospital, Egerton Road, Guildford, GU2 5XX, UK

**Keywords:** squamous cell cancer of the head and neck, *CDKN2A* (*p16*) gene, mutation

## Abstract

There is increasing evidence that predisposition to some cancers has a genetic component. There is a high incidence of loss of heterozygosity on chromosome 9, in the region of tumour suppressor gene *CDKN2A* (also known as *p16*), in sporadic squamous cell cancer of the head and neck (SCCHN). To investigate the possibility that *CDKN2A* may be involved in the inherited susceptibility to SCCHN, the 3 coding exons of *CDKN2A* were sequenced in 40 patients who had developed a second primary cancer after an index squamous cell cancer of the head and neck. No mutations were found and we conclude that *CDKN2A* mutations do not play a major role in cancer susceptibility in this group. © 2001 Cancer Research Campaign


*British Journal of Cancer* (2001) **85**(9), 1383–1386
© 2001 Cancer Research Campaign
doi: 10.1054/ bjoc.2001.2068, available online at http://www.idealibrary.com on IDEAL

http://www.bjcancer.com

### No germline mutations in *CDKN2A* (*p16*) in patients with squamous cell cancer of the head and neck and second primary tumours

**S Jefferies[1], SM Edwards[1], RA Hamoudi[1], R A'Hern[2], W Foulkes[3], D Goldgar[4], MPT Collaborators* and R Eeles[1/2]**

[1]Cancer Genetics, Institute of Cancer Research, 15 Cotswold Rd, Sutton, Surrey; [2]The Royal Marsden Hospital Trust, Downs Rd, Sutton, Surrey, SM2 5PT; [3]McGill University, Montreal, Canada; [4]IARC, Lyon, France; [6]Guys and St Thomas' and Kings Hospital Trusts; [7]Mount Vernon Hospital Trust, Rickmansworth Road, Northwood, Middlesex, HA6 2RN; [8]Mid-Kent Oncology Centre, Hermitage Lane, Maidstone, Kent, ME16 9QQ; [9]Queen Victoria Hospital, East Grinstead; [10]Royal Surrey County Hospital, Egerton Road, Guildford, GU2 5XX, UK

**Summary** There is increasing evidence that predisposition to some cancers has a genetic component. There is a high incidence of loss of heterozygosity on chromosome 9, in the region of tumour suppressor gene, *CDKN2A* (also known as *p16*), in sporadic squamous cell cancer of the head and neck (SCCHN). To investigate the possibility that *CDKN2A* may be involved in the inherited susceptibility to SCCHN, the 3 coding exons of *CDKN2A* were sequenced in 40 patients who had developed a second primary cancer after an index squamous cell cancer of the head and neck. No mutations were found and we conclude that *CDKN2A* mutations do not play a major role in cancer susceptibility in this group. © 2001 Cancer Research Campaign

**Keywords**: squamous cell cancer of the head and neck; *CDKN2A* (*p16*) gene; mutation

Squamous cell cancer of the head and neck (SCCHN) is a disease associated with major morbidity and mortality. It is the fifth most common cancer worldwide and the incidence of SCCHN is increasing in developing countries (Macfarlane et al, 1994; Parkin et al, 1985). The epidemiology of SCCHN is complex due to the multigenic nature of the disease and the number of potential environmental agents to which individuals may have been exposed. It is clear that the major aetiological agents are tobacco and alcohol exposure (Maier et al, 1992; Vokes et al, 1993). Alcohol and tobacco exposure increase the risk of SCCHN in a dose-dependent and independent manner. Other risk factors include nutrition, occupation, viral infection and poor dentition (Maier et al, 1991; Muscat and Wynder, 1992). These risk factors do not, however, adequately explain all cancer cases. Until recently, little attention has been paid to possible hereditary factors in SCCHN. However, 3 recent case–control studies have shown a relative risk (RR) for SCCHN of 3.5–3.8 in association with a family history of cancer of the upper aerodigestive tract (UADT) and/or lung cancer (Copper et al, 1995; Foulkes et al, 1995, 1996).

Despite therapeutic advances in the treatment of SCCHN, the overall survival from the disease has remained unchanged over the last 20 years. A contributory factor to this is that patients with successfully treated early-stage SCCHN have a high incidence of second primary cancers of SCCHN or UADT (Day and Blot, 1992). Generally, the presence of multiple primary cancers in an individual is a sign that there may be an increased hereditary predisposition to cancer in that person. Inter-individual differences in susceptibility to second tumours could be accounted for by genetic factors (Trizna and Schantz, 1992; Bongers et al, 1996). Individuals with a second primary SCCHN are more likely to have a family history of SCCHN than those with only single primaries (Bongers et al, 1996; Foulkes et al, 1996; Morita et al, 1998).

The tumour suppressor gene *CDKN2A* (also known as *p16*) is localized on chromosome 9p21 and is thought to play an important role in the development of some cancers. The gene consists of 3 coding exons, exon 1 (125 bp), exon 2 (307 bp) and exon 3 (12 bp) (Kamb et al, 1994a). It encodes a 16 kDa protein that binds to cyclin-dependent kinase (cdk) 4 and 6 inhibiting their association with cyclin D1 (Serrano et al, 1993). The inhibition of the cyclin D1-cdk4/6 complex activity prevents retinoblastoma protein phosphorylation, which normally leads to the arrest of the cell cycle in the G1/S transition (Serrano et al, 1993). Somatic *CDKN2A* deletions have been documented in melanoma, oesophageal, lung and bladder cancer (Kamb et al, 1994b). Germline *CDKN2A* mutations have been shown to predispose to familial melanoma (Hussussian et al, 1994; Kamb et al, 1994b). *CDKN2A* is thought to be an important tumour suppressor gene in the aetiology of SCCHN. Somatic mutations occur in 10% of SCCHN and homozygous deletions occur in approximately 50% (Reed et al, 1996). Inactivation of *CDKN2A* is thought to be an early event in the development of SCCHN. In families with familial melanoma, an association has been reported with SCCHN and pancreatic carcinoma (Whelan et al, 1995). In one study of 67 cancer prone families, a germ-line mutation in *CDKN2A* (M531) was identified in a woman with multiple primary tumours, including SCCHN. Her grandmother was reported to have an oral canncer (Sun et al, 1997).

The aim of this study was to investigate whether germline *CDKN2A* mutations contributed to the postulated increased

*Received 13 June 2000*
*Revised 16 July 2001*
*Accepted 23 July 2001*

*Correspondence to:* S Jefferies

*MPT Collaborators: J Henk[2], M Gore[2], P Rhys-Evans[2], D Archer[2], K Bishop[2], E Solomon[6], S Hodgson[6], M McGurk[6], J Hibbert[6], M O'Connell[6], M Partridge[6], E Chevretton[6], F Calman[6], M Saunders[7], K Shotton[9], A Brown[9], S Whittaker[10].

susceptibility of tumour development amongst patients who had an index SCCHN followed by a second cancer.

### MATERIALS AND METHODS

### Patient selection

Individuals with index SCCHN who had developed a second primary cancer in accordance with the criteria defined by Hong et al (1990) were identified for the study. An SPT is defined as occurring more than 3 years from the original diagnosis or is separated by greater than 2 cm of entirely normal epithelium. If the SPT occurs in the lung it must be solitary and occur greater than 3 years from the original diagnosis. If an SPT is of different histology these criteria do not apply. An SPT can occur anywhere and is not just limited to the UADT. Venous blood samples in EDTA were obtained from 40 patients with histologically proven SCCHN and an SPT. The patients were participants in a multicentre molecular epidemiology study assessing the possible underlying genetic mechanisms in head and neck cancer. Clinical and epidemiological characteristics were recorded. A detailed first-degree family history pedigree was obtained from each participant. All blood samples were obtained with written informed consent and ethical approval from participating centres.

### Patient characteristics

24 of the patients were male and 16 female. The mean age at initial presentation was 61.3 years (+/–9.8). All had histologically proven SCCHN as an index tumour. The predominant primary site was oral cavity (22 patients) followed by larynx (12), hypopharynx (4) and oropharynx (2). Of the second cancers, 24 were SCCHN, 7 prostate adenocarcinoma, 3 lung SCC, 2 oesophageal SCC, 2 melanoma, 1 colon adenocarcinoma and 1 chronic granulocytic leukaemia. 3 patients had 3 primaries in total; 2 had further SCCHN and 1 developed adenocarcinoma of the lung.

### DNA extraction

Peripheral blood samples (20 ml) were collected from 40 patients with SPT and stored in EDTA at –70˚C. DNA extraction was undertaken by the method of Kunkel et al (1977) with some modifications. 4 volumes of water were initially added to whole blood. The phenol-chloroform extraction step was not included and the salting out procedure of Miller et al (1988) was used to clean and retrieve the DNA. DNA was washed in 70% alcohol and dried before dissolving in 0.3 ml of water.

### Polymerase chain reaction (PCR)

Sample DNA was added to a reaction mix containing 10X buffer (Perkin Elmer), 1.5 mmol $MgCl_2$ for exon 1 and 2, 1.25 mmol for exon 3, 0.8 mM dNTPs, 1.0 µM primer and 0.4 µl of AmpliTaq Gold (Perkin Elmer), and 5% DMSO. The primer sequences used were as described by Kamb et al (1994b) and Hussussian et al (1994). The total reaction was made up to 50 µl with water. The tubes were topped with mineral oil (Sigma) and cycled in a Hybaid thermocycler. Thermocycling for exon 1 and 2 was 95˚C for 9 min; 94˚C for 1 min, 60–55˚C for 1 min touchdown, 72˚C for 1 min – 11 cycles; 94˚C for 1 min, 55˚C for 1 min, 72˚C for 1 min – 25 cycles; 72˚C for 10 min. Thermocycling for exon 3 was 95˚C for 5 min; 95˚C for 1 min, 55–50˚C for 1 min touchdown, 72˚C for 1 min – 11 cycles; 95˚C for 1 min, 50˚C for 1 min, 72˚C for 1 min – 34 cycles; 72˚C for 10 min (adapted from Harland et al 1997). A 5 µl aliquot of PCR reaction mix was run on 2% agarose gel to check for presence of the required product. PCR products were purified before dye terminator sequencing according to Hamoudi et al 1996. The DNA was dissolved in 15 µl of water and an aliquot was run on 2% agarose gel to check presence and purity of the product. A positive control was included in the sample set, a known mutation in exon 3 (supplied by Harland et al, 1997).

### Cycle sequencing

PCR products were sequenced directly using an ABI prism dye terminator cycle sequencing reaction kit. Thermocycling was 96˚C for 1 min; 96˚C for 30 s, 50˚C for 15 s, 60˚C for 60 s for 25 cycles. The extension products were added to 3 M sodium acetate, precipitated with absolute alcohol and centrifuged. The pellet was washed with 70% alcohol, dried and then stored at –20˚C.

### Automated sequencing

Exons 1, 2 and 3 of CDKN2A were sequenced in both the forward and reverse direction. Sequencing was conducted on a 6% polyacrylamide denaturing gel in a 1X TBE (Tris borate buffer) using an ABI 377 automated flourescent DNA sequencer. The DNA pellet was dissolved in 4 µl of formamide and loading dye. The data were stored using DNA Sequencing Analysis Software. On completion of the run the data were analysed using Sequence Navigator software (ABI).

### RESULTS AND DISCUSSION

Evidence is increasing that inter-individual differences in cancer susceptibility may be genetically determined. This genetic component in diseases such as SCCHN is likely to be multifactorial and will include alterations in germline tumour suppressor genes, metabolic enzyme function and DNA repair mechanisms. Second primary tumours occur in 10–30% of patients with SCCHN (Franco et al, 1991). As second or multiple primary tumours are associated with several of the hereditary cancer syndromes, Li–Fraumeni syndrome, Bloom syndrome and Fanconi anaemia, (Trizna and Schantz, 1992) and family studies have demonstrated an increased risk of SCCHN amongst relatives of patients with SPT (Bongers et al, 1996; Foulkes et al, 1996; Morita et al, 1998) we assessed the possible role of germline *CDKN2A* mutations in the development of SPT.

Further support for this hypothesis is that *CDKN2A* alterations are common in human cancer and high frequencies of somatic homozygous deletions and mutations are seen in SCCHN (Reed et al, 1996; Miracca et al, 1999). Melanoma kindreds with CDKN2A mutations have also been described with cases of SCCHN (Lynch et al, 1981; Whelan et al, 1995; Yarborough et al, 1996). We screened the full coding sequence of *CDKN2A* for germline mutations in 40 patients with index SCCHN and an SPT. No mutations were identified within the coding region of the gene (incidence 0%: 95% CI 0–8%). A control mutation in exon 3 was detected. One patient was found to have a c→g transition (+32), not previously described, in exon 3. These results suggest that germline mutations in *CDKN2A* do not contribute to the increased cancer susceptibility in this group.

 *© 2001 Cancer Research Campaign*

There are a number of issues to consider from the results of this study. In the methodology used, we cannot exclude the possibility that we may have missed a small number of mutations within the *CDKN2A* gene. We would also not have detected changes in the promoter region or introns of *CDKN2A*, which in some way might abrogate the function of the gene. Germline mutations in *CDKN2A* are responsible for the predisposition to melanoma in some families, but not all families that demonstrate 9p21 linkage are found to have mutations. 2 studies of mutation-negative families have demonstrated a 5′ UTR variant (G-34T) which creates an aberrant initiation codon (Liu et al, 1999; Harland et al, 2000). This alteration and other non-coding mutations may be important in a small percentage of families.

The selection of patients may have led to a number of factors that could have obscured an underlying genetic effect. Patients were identified for this study who had developed index SCHNN and then an SPT. This resulted in a heterogeneous population in terms of the primary site within the head and neck and subsequent site of the second primary cancer. It is known that younger age of onset of cancer may be associated with an underlying genetic predisposition. However, in this study the average age of development of SCCHN was 61.3 years (+/–9.8 years), which was not lower than observed in a series of single incident cases of SCCHN (62.2 years: SD +/– 13) (data not shown). A family history of cancer is also a marker for possible genetic predisposition. 27 patients had a family history of cancer of which 4 had a relative with SCCHN (Table 1). The male–female ratio was 3:2, which is a higher proportion of females than would be expected in incident single SCCHN, and may reflect gender-based differences in predisposition to second cancers.

The definition of second primary tumours in this study was based on the clinical definition defined by Hong et al (1990). It is likely that some of the patients included in this study had in fact developed a metastasis from their index tumour, and are not genetically predisposed to SPT. Molecular studies have demonstrated solitary lung tumours to be metastases rather than new primaries (Leong et al, 1998) and similarly synchronous and metachronous lesions have been shown to be genetically identical (Worsham et al, 1995; Bedi et al, 1996; Scholes et al, 1998). Results from molecular studies may be incorporated in the definition of SPT in the future.

Other studies assessing patients with multiple primary tumours have been successful in demonstrating germline mutations of tumour suppressor genes. Germline *p53* has been identified in patients with multiple primary tumours and members of cancer-prone families (Wang et al, 1996). Gallo et al (1999) screened 24 consecutive patients with index SCCHN and SPT for *p53* mutations. *p53* germline mutations were identified in the peripheral blood and cancers of 3 patients, 1 was a missense mutation in exon 6 and the other 2 were same-sense polymorphisms with no change in the amino acid sequence of the p53 protein. Abnormal expression of wild-type p53 protein was seen in normal and pathological tissue from the 2 patients with the p53 polymorphisms.

Another tumour suppressor gene that has been assessed in SCCHN is the *BRCA2* gene. Easton et al (1997) reported an excess of laryngeal cancer (relative risk = 7.67) in a large hereditary breast cancer family. A significant number of cancers of the buccal cavity and pharynx were observed in a study examining a large series of *BRCA2* families from North America and Europe (RR = 2.26; *P* = 0.03) (Breast Cancer Linkage Consortium, 1999). Cancers of the throat and oral cavity were also observed in *BRCA2* carriers in 3 out of 17 French Canadian breast cancer families (Tonin et al, 1998). Hamel et al (1999) assessed 78 cases of SCCHN for founder mutations in *BRCA2* (53 were French Canadian; 25 Ashkenazi Jewish cases). No founder *BRCA2* mutations were observed and therefore the study concluded that screening for *BRCA2*-inherited mutations, as a risk factor for SCCHN, is not warranted.

Although mutations in the *CDKN2A* gene have been reported in kindreds with oral cancer and familial melanoma, we have failed to demonstrate any coding germline *CDKN24* mutations in 40 patients presenting with index SCCHN and subsequent second primary tumours. Other mechanisms that may increase cancer susceptibility in this group, such as metabolic enzyme polymorphisms and DNA repair enzyme polymorphisms and their relationship with smoking and alcohol exposure are currently being explored.

**Table 1** Age at diagnosis and family history (FH) of SPT patients

| Sex | Male (*n* = 24) | Female (*n* = 16) |
|---|---|---|
| Mean age | years (+/–SD) | |
| All (*n* = 40) | 61.3 (9.8) | |
| FH SCCHN (*n* = 4) | 68.5 | |
| FH CA (*n* = 23) | 59.7 | |
| No FH (*n* = 3) | 61.9 | |

### ACKNOWLEDGEMENTS

S Jefferies is supported by a Cancer Research Campaign Fellowship for Clinicians. We thank the patients for their participation in this study.

### REFERENCES

Bedi G, Westra W, Gabrielson E, Koch W and Sidransky D (1996) Multiple head and neck tumours: evidence for clonal origin. *Cancer Res* **56**: 2484–2487

Bongers V, Braakhuis B, Tobi H, Lubson H and Snow GB (1996) The relation between cancer incidence among relatives and the occurrence of multiple primary carcinomas following head and neck cancer. *Cancer Epidemiol Biomarker Prev* **5**: 595–598

Copper M, Jovanic A, Nauta JJ, Braakhuis BJM, de Vries N, van der Waal I and Snow GB (1995) Role of genetic factors in the aetiology of squamous cell carcinoma of the head and neck. *Arch Otolaryngol Head Neck Surg* **121**: 157–160

Day GL and Blot WJ (1992) Second primary tumours in patients with oral cancer. *Cancer* **70**: 14–19

Easton DF, Steele L, Fields P, Ormiston W, Averill D, Daly PA, McManus R, Neuhausen SL, Ford D, Wooster R, Cannon-Albright LA, Stratton MR and Goldgar DE (1997) Cancer risks in two large breast cancer families linked to BRCA2 on chromosome 13q12–13. *Am J Hum Genet* **61**: 120–128

Franco EL, Kowalski LP and Kand JL (1991) Risk factors for second cancers of the upper respiratory and digestive systems: a case-control study. *J Clin Epidemiol* **44**: 615–625

Foulkes WD, Brunet J-S, Kowalski LP, Narod SA and Franco EL (1995) Family history is a risk factor for squamous cell carcinoma of the head and neck in Brazil: a case-control study. *Int J Cancer* **63**: 769–773

Foulkes WD, Brunet J-S, Sieh W, Black MJ, Shenouda G and Narod SA (1996) Familial risk of squamous cell carcinoma: a retrospective case-control study. *BMJ* **313**: 716–721

Gallo O, Sardi I, Pepe G, Franchi A, Attanasio M, Giusti B, Bocciolini C and Abbate R (1999) Multiple primary tumours of the upper aerodigestive tract: is there a role for constitutional mutations in the p53 gene? *Int J Cancer* **82**: 180–186

© 2001 Cancer Research Campaign 

Hamel N, Manning A, Black MJ, Tonin PN and Foulkes WD (1999) An absence of founder BRCA2 mutations in individuals with squamous cell carcinoma of the head and neck. *Int J Cancer* **83**: 803–804

Hamoudi RA, De Schouwer PJ and Yuille MA (1998) Improved direct flourescent automated sequencing of PCR products. *Trends Genet* **1**: 1–2

Harland M, Meloni R, Nelleke G, Pinney E, Brookes S, Spurr K, Frischauf A-M, Bataille V, Peters G, Cuzick J, Selby P, Bishop DT and Bishop JN (1997) Germline mutations of the CDKN2A gene in UK melanoma families. *Human Molecular Genetics* **6**: 2061–2067

Harland M, Holland EA, Ghorizo P, Mantelli M, Bianchi-Scarra G, Goldstein AM, Tucker MA, Ponder BA, Mann GJ, Bishop DT and Bishop JN (2000) Mutation screening of the CDKN2A promoter in melanoma families. *Genes Chromosomes Cancer* **28**: 45–57

Hong WK, Lippman SM, Itri LM, Karp DD, Lee JS and Byers RM (1990) Prevention of second primary tumours with isoretinionin squamous-cell carcinoma of the head and neck. *New Eng J Med* **323**: 795–801

Hussussian CJ, Struewing JP, Goldstein AM, Higgins PA, Ally DS, Sheahan MD, Clark WH, Tucker MA and Dracopoli NC (1994) Germline p16 mutations in familial melanoma. *Nature Genetics* **8**: 15–21

Kamb A, Gruis NA, and Weaver-Feldhaus J, Liu Q, Harshman K, Tavtigian SV, Stockert E, Day RS 3rd, Johnson BE and Scolnick MH (1994a) A cell cycle regulator potentially involved in the genesis of many tumour types. *Science* **264**: 436–437

Kamb A, Shattuck-Eidens D, Eeles R, Liu Q, Gruis NA, Ding W, Hussey C, Tran T, Miki Y, Weaver Feldhaus et al (1994b) Analysis of the p16 gene as a candidate for the chromosome 9p melanoma susceptibility locus. *Nat Genet* **8**: 22–26

Liu L, Dilworth D, Gao L, Monzon J, Summers A, Lassam N and Hogg D (1999) Mutation of the CDKN2A 5′ UTR creates an aberrant initiation codon and predisposes to melanoma. *Nat Genetics* **21**: 128–132

Leong P, Banafsheh R, Koch W, Reed A, Eisele D, Lee DJ, Sidransky D, Jen J and Westra W (1998) Distinguishing second primary cancers from lung metastases in patients with head and neck cancer. *J Natl Cancer Inst* **90**: 972–977

Lynch H, Fusaro R, Pester J, Oosterhuis J, Went L and Rumke P (1981) Tumour Spectrum in the FAMM syndrome. *Br J Cancer* **44**: 553–560

Kunkel LM, Smith KD and Boyer SH (1977) Analysis of human Y-chromosome specific reiterated DNA in chromosome variants. *Proc Natl Acad Sci* **74**: 1245–1249

Macfarlane G, Boyle P, Evstifeeva T, Robertson C and Scully C (1994) Rising trends of oral cancer mortality among males worldwide; the return of an old public health problem. *Cancer Causes Control* **5**: 259–265

Maier H, Dietz A, Gewelke U and Heller WD (1991) Occupational exposure to hazardous substances and risk of cancer of the oral cavity, hypopharynx and larynx. *Laryngol Rhinol Otol* **70**: 93–98

Maier H, Dietz A, Gewelke U, Heller WD and Weidauer H (1992) Tobacco, alcohol and the risk of head and neck cancer. *Clin Invest* **70**: 320–327

Miller SA, Dykes DD and Polesky HF (1988) A simple salting out procedure for extracting DNA from human nucleated cells. *Nucleic Acids Res* **16**: 1215

Miracca E, Kowalski L and Nagai M (1999) High prevalence of p16 genetic alterations in head and neck tumours. *Br J Cancer* **81**(4): 677–683

Morita M, Kuwano H, Nakashima T, Taketomi A, Baba H, Saito T, Tomoda H, Egashir H, Kawaguchi H, Kitamura K and Sugimachi K (1998) Family aggregation of carcinoma of the hypopharynx and cervical oesophagus: special reference to multiplicity of cancer in the upper aerodigestive tract. *Int J Cancer* **76**: 468–471

Muscat J and Wynder E (1992) Tobacco, alcohol, asbestos and occupational risk factors for laryngeal cancers. *Cancer* **69**: 2244–2251

Parkin DM, Pisani P and Ferlay J (1995) Estimates of the world-wide incidence of eighteen major cancers in 1995. *Int J Cancer* **54**: 594–606

Reed A, Califano J, Cairns P, Westra W, Jones R, Koch W, Ahrendt S, Eby Y, Sewell D, Nawroz H, Bartek J and Sidransky D (1996) High frequency of p16 inactivation in head and neck squamous cell carcinoma. *Cancer Res* **56**: 3630–3633

Scholes A, Woolgar J, Boyle , Brown JS, Vaughn ED, Hart CA, Jones AS and Field JK (1998) Synchronous oral cancer: independent or common clonal origin. *Cancer Res* **58**: 2003–2006

Serrano M, Hannon G and Beach D (1993) A new regulatory motif in cell cycle control causing specific inhibition of cyclin D/CDK4. *Nature* **366**: 704–707

Sun S, Pollock P, Liu L, Karimi S, Jothy S, Milner B, Renwick A, Lassam N, Hayward N, Hogg D, Narod S and Foulkes W (1997) *CDKN2A* mutation in a non-FAMM kindred with cancers at multiple sites results in a functionally abnormal protein. *Int J Cancer* **73**: 531–536

Tonin PN, Mes-Masson AM, Futreal PA, Morgan K, Mahon M, Foulkes WD, Cole DE, Provencher D, Ghadirian P and Narod SA (1998) Founder BRCA1 and BRCA2 mutations in French Canadian breast and ovarian cancer families. *Am J Hum Genet* **63**: 1341–1351

Trizna Z and Schantz SP (1992) Hereditary and environmental factors associated with risk and progression of head and neck cancer. *Otolaryngol Clin North Am* **25**: 1089–1103

Vokes EE, Weichselbaum RR, Lippman SM and Hong WK (1993) Head and Neck Cancer. *N Eng J Med* **328**: 184–194

Wang Q, Lasset C, Sobol H and Ozturk M (1996) Evidence of a hereditary p53 syndrome in cancer-prone families. *Int J Cancer* **65**: 554–557

Whelan A, Bartsch D and Goodfellow P (1995) Brief report: a familial syndrome of pancreatic cancer and melanoma of pancreatic cancer and melanoma with a mutation in the CDKN2 tumour-suppressor gene. *N Engl J Med* **333**: 975–977

Worsham M, Wolman S, Carey T, Zarbo RJ, Benninger MS and Van Dyke DL (1995) Common clonal origin of synchronous primary head and neck squamous cell carcinomas: analysis by tumour karyotype and FISH. *Hum Pathol* **26**: 251–261

Yarborough W, Aprelikova O, Pei H, Olshan A and Liu E (1996) Familial Tumour Syndrome Associated With a Germline Nonfunctional p16INK4a Allele. *JNCI* **88**(20): 1489–1491

*© 2001 Cancer Research Campaign*
